# Adverse Drug Reaction Discovery Using a Tumor-Biomarker Knowledge Graph

**DOI:** 10.3389/fgene.2020.625659

**Published:** 2021-01-12

**Authors:** Meng Wang, Xinyu Ma, Jingwen Si, Hongjia Tang, Haofen Wang, Tunliang Li, Wen Ouyang, Liying Gong, Yongzhong Tang, Xi He, Wei Huang, Xing Liu

**Affiliations:** ^1^School of Computer Science and Engineering, Southeast University, Nanjing, China; ^2^Department of Pharmaceutical Sciences, Tsinghua University, Beijing, China; ^3^Department of Anesthesiology, Third Xiangya Hospital, Central South University, Changsha, China; ^4^College of Design and Innovation, Tongji University, Shanghai, China; ^5^Department of Intensive Care Unit, Third Xiangya Hospital, Central South University, Changsha, China; ^6^Department of Cardiology, Third Xiangya Hospital, Central South University, Changsha, China

**Keywords:** adverse drug reaction, biomarker, knowledge graph, antitumor drugs, explainable model

## Abstract

Adverse drug reactions (ADRs) are a major public health concern, and early detection is crucial for drug development and patient safety. Together with the increasing availability of large-scale literature data, machine learning has the potential to predict unknown ADRs from current knowledge. By the machine learning methods, we constructed a Tumor-Biomarker Knowledge Graph (TBKG) which contains four types of node: Tumor, Biomarker, Drug, and ADR using biomedical literatures. Based on this knowledge graph, we not only discovered potential ADRs of antitumor drugs but also provided explanations. Experiments on real-world data show that our model can achieve 0.81 accuracy of three cross-validation and the ADRs discovery of Osimertinib was chosen for the clinical validation. Calculated ADRs of Osimertinib by our model consisted of the known ADRs which were in line with the official manual and some unreported rare ADRs in clinical cases. Results also showed that our model outperformed traditional co-occurrence methods. Moreover, each calculated ADRs were attached with the corresponding paths of “tumor-biomarker-drug” in the knowledge graph which could help to obtain in-depth insights into the underlying mechanisms. In conclusion, the tumor-biomarker knowledge-graph based approach is an explainable method for potential ADRs discovery based on biomarkers and might be valuable to the community working on the emerging field of biomedical literature mining and provide impetus for the mechanism research of ADRs.

## Introduction

Adverse drug reactions (ADRs) are a cause of significant morbidity and mortality in patients and a source of financial burden in the healthcare system (Patton and Borshoff, 2018). In tumor patients, pharmacokinetic parameters can be altered by the disease itself, or hepatic or renal impairment, or reduction of serum-binding proteins due to malnutrition. They experience a relatively high rate of ADRs from antitumor drugs and more easily experience rare and severe ADRs, which could seriously impact the quality of life (Shrestha et al., 2017). The identification of rare and serious ADRs during the premarket period is limited due to the limited sample size and generalizability of clinical trials. Exploring the potential ADRs is critical to decrease the incidence. Therefore, great efforts have been devoted to detecting potential ADRs based on the data mining of literature databases or electronic health records (Bean et al., 2017; Lee and Chen, 2020). However, there are still two challenges to achieving good performance: (1) the unstructured biomedical literature contains many irrelevant words and contexts, and how to extract ADR-related entities and fully explore their relations (e.g., tumor-biomarker-drug) is difficult; and (2) some predicted unseen ADRs are unexpected and cause confusion, which means explainability and validation become critically important for automatic detection.

**Figure 1:**
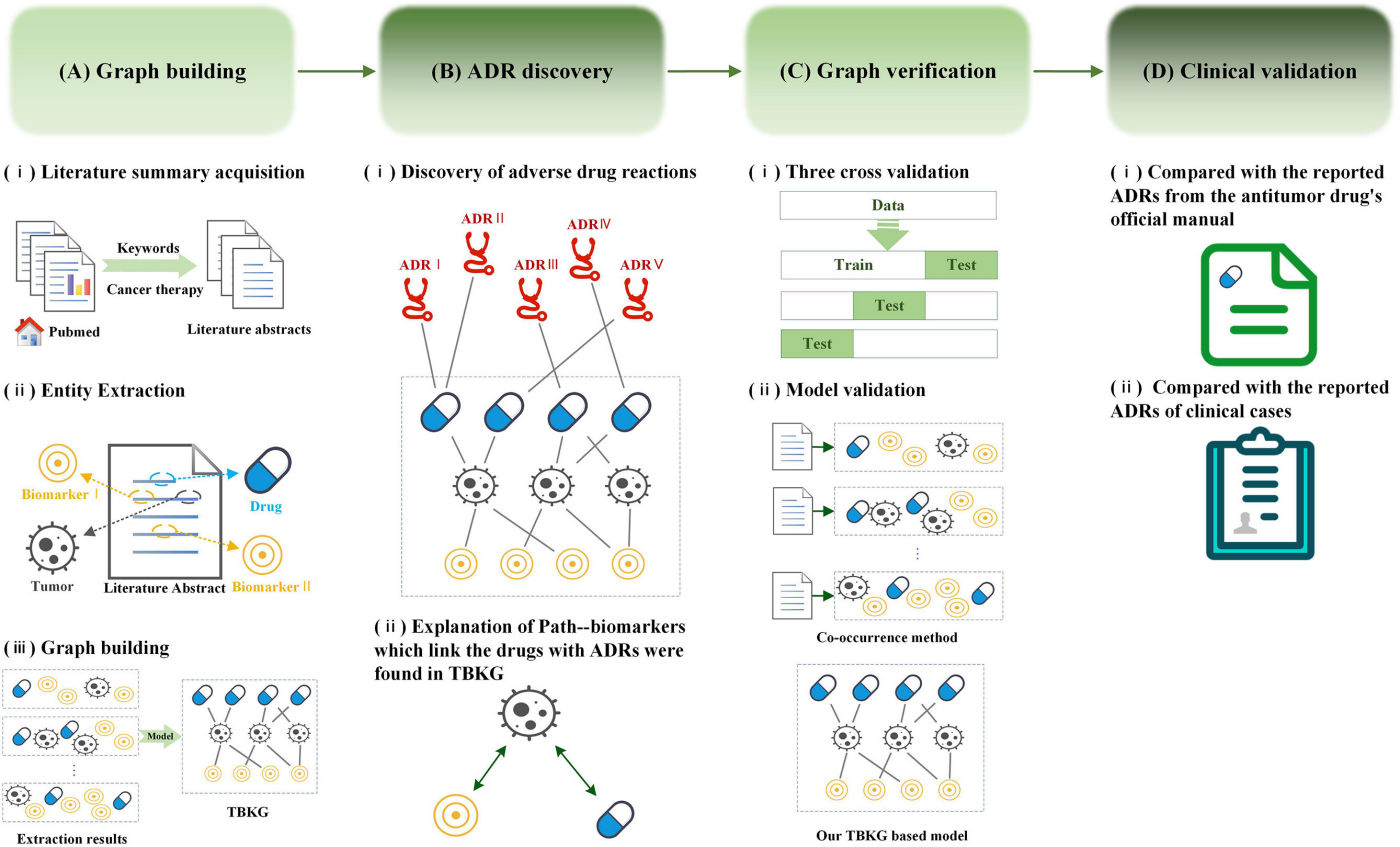
Workflow for adverse drug reaction discovery using the Tumor-Biomarker Knowledge Graph (TBKG). **(A)** The construction process of TBKG. **(B)** The results of ADR discovery based on TBKG. **(C)** Preclinical verification of the ADR discovery based on TBKG. **(D)** Clinical validation of the ADR discovery based on TBKG.

A knowledge graph (Wang et al., 2017a,b) is a data model that represents facts as nodes and relations between the nodes. Under a general medical information network, objects such as diseases, drugs, biomarkers, or treatments can all be linked together through different types of referential relationships, which enable the discovery of knowledge on a scale and at a speed that traditional pharmacologic experiments or clinical trials cannot approach. Recently, in addition to diagnosis and prognostication, biomarker (Califf, 2018; Carr and Pirmohamed, 2018) has been widely in tumor treatment to offer the opportunity to accurately and specifically predict therapeutic efficacy and safety during the course of antitumor therapy, which can provide oncologists with the opportunity to quickly modify a therapeutic regimen in ways that would provide the best therapy for their patients. However, there is little biomarker beginning to be applied to assess the ADRs. The aim of this study is to discover potential ADRs of anti-tumor drugs and provide explanations by constructing knowledge graph using literature data source. Figure 1 summarizes the workflow of our study.

## Methods

### Data Source

The biomedical database employed in this study was the MEDLINE database, which consists of more than 22 million journal citations and abstracts. The database is maintained by the National Library of Medicine (NLM). The MEDLINE corpus can be acquired -in XML format from http://www.nlm.nih.gov/bsd/licensee/access/medline_pubmed.html. Each citation contains the bibliographical information of an article, such as the article ID (PubMed Unique Identifier, PMID), article title, author list, journal title, venue, publication type, and indexed MeSH terms. MEDLINE is used as a surrogate for full-text articles. Permission to access the data were acquired by the 3rd Xiangya Hospital in China in May 2016.

The Unified Medical Language System (UMLS) Metathesaurus integrates the information of 216 source vocabularies and brings together many different types of biomedical vocabularies, mainly including 25% diagnosis, 25% procedures and supplies, 19% diseases and 14% drugs. Metathesaurus refines these categories to 127 different categories. As shown in Table 1, the category description of T191 is “Neoplastic Process,” therefore, we fit it into tumor-type nodes. The category descriptions of T121 and T200 are “Pharmacologic Substance” and “Clinical Drug,” respectively, we hence fit it into drug-type nodes. For ADR-type nodes, we use the WHO source dictionary in UMLS because WHO is used for coding clinical information related to adverse drug reactions (Supplementary Table 1). Finally, for biomarker-type nodes, there are many types of biomarker referring to the definition of biomarker (Carr and Pirmohamed, 2018), including genomic, immunogenetic, circulating protein, nucleic acid and so on. Therefore, we fit the corresponding categories T109, T114, T116, T123, T125, T126, T129, T130, T192, and T195 in Table 1 into biomarker-type nodes.

**Table 1:** The concept category used to build the dictionary.

**Specific categories**	**Category meaning**	**Type in TBKG**
T109	Organic Chemical	Biomarker-type
T114	Nucleic Acid, Nucleoside, or Nucleotide	Biomarker-type
T116	Amino Acid, Peptide, or Protein	Biomarker-type
T121	Pharmacologic Substance	Drug-type
T123	Biologically Active Substance	Biomarker-type
T125	Hormone	Biomarker-type
T126	Enzyme	Biomarker-type
T129	Immunologic Factor	Biomarker-type
T130	Indicator, Reagent, or Diagnostic Aid	Biomarker-type
T191	Neoplastic Process	Tumor-type
T192	Receptor	Biomarker-type
T195	Antibiotic	Biomarker-type
T200	Clinical Drug	Drug-type

### Tumor-Biomarker Knowledge Graph-based ADR Discovery

#### Entity Extraction

From MEDLINE, we downloaded the abstracts of papers from 1928 to 2020 with “cancer therapy” as the key word. The article number, title, author, author unit, publication time, MeSH word, journal title and publication type were saved, and short abstracts were removed.

Four kinds of entities were extracted from the abstracts: tumors, biomarkers, drugs, and ADRs. We used the Metathesaurus 2020AA version provided by UMLS as the dictionary for entity extraction. Apache's open source tool cTAKES, which is a natural language processing system for extracting information from medical free texts, was utilized to extract entities. An entity mentioned positively was seen as an entity that was related to each abstract. Here, we limited the minimum frequency of entities to 50.

#### Relation Discovery

**Figure 2:**
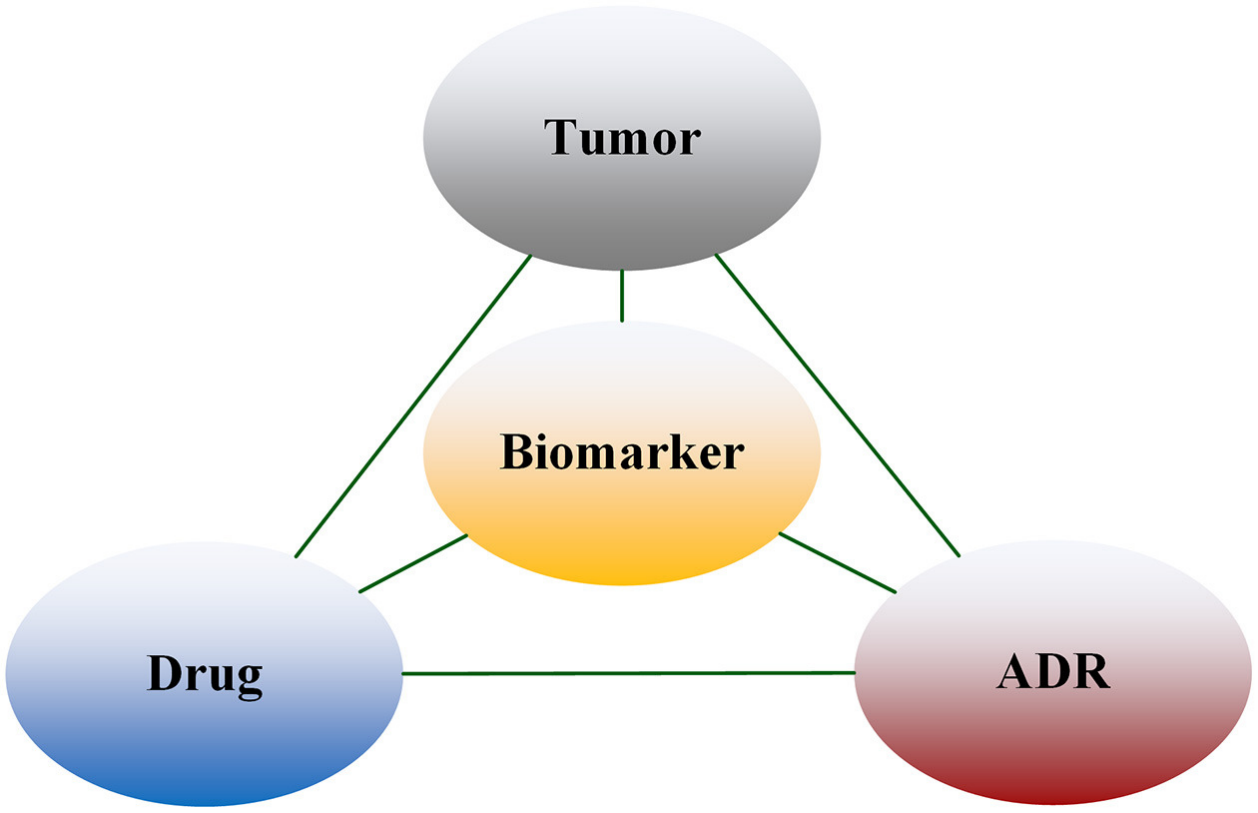
Structure of TBKG.

We used the entities extracted above to construct the Tumor-Biomarker Knowledge Graph (TBKG). TBKG is defined as G = (V, E), where G stands for TBKG, V is the set of vertices in G, and E is the set of edges in G. V contains the vertices of four entity types, namely tumor, biomarker, drug, and ADR. E contains undirected weighted edges. Each edge connects two different types of vertices. The weight on the edge represents the correlation (distance) between the two vertices. A basic schema is shown in Figure 2. Entities were transformed into matrix form. Each row of the matrix represented a summary file, and whether the entity appeared in the summary was represented by 0 and 1; this matrix data was taken as the input of the model.

**Figure 3:**
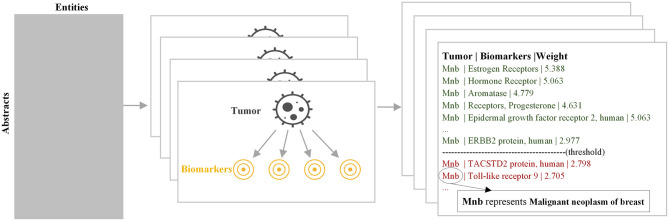
Workflow of modeling the relationship between diseases and biomarkers.

A naive Bayesian model (Murphy, 2012) was utilized to explore correlations. The naive Bayesian model combined the prior probability and the posterior probability at the same time when building the graph, which could avoid the subjective bias from using only the prior probability and avoided the overfitting phenomenon from using the sample information alone at the same time. The calculation method for each relationship was the same, and we mainly used the calculation of the relationship between tumor and biomarker to illustrate the principle of this model. The parameter was learned by Maximum Likelihood Estimation. We learned a model for each tumor, as shown in Figure 3. An importance measure was used to determine whether there was a relationship between the tumor and biomarker:

(1) $$\begin{array}{r} IMPT_{NB}=\log\left(p\left(x_{i}=1|y_{j}=1\right)\right)-\log\left(p\left(x_{i}=1|y_{j}=0\right)\right), \end{array}$$

where *x*_*i*_ is 0 or 1 to indicate the presence of biomarker *i* and *y*_*j*_ is 0 or 1 to indicate the presence of tumor *j*. The reason why we use the importance measure is that if the presence of a biomarker makes it more likely that a tumor will be observed, we are more confident that there is a relationship between them. Relationship whose importance was greater than a certain threshold was considered to exist.

#### ADR Discovery

For the ADR discovery based on TBKG, we collected all drugs and determined the corresponding ADR to form drug-ADR pairs as the calculated ADRs. The Depth First Search (DFS) algorithm was utilized to find every path between the drug and ADR, such as (drug, biomarker, ADR). Each output of ADR discovery contains a drug-ADR pair and all corresponding paths.

### Experimental Settings

#### Accuracy With Cross-Validation

Three-fold cross-validation, which is mainly used to prevent overfitting caused by the model being too complex, was utilized to verify the effect of graph construction. The basic idea is to divide the original data into a training set to train the model and a test set to test the training results. The original data were randomly divided into three groups and each time, two groups were selected as the training set and the remaining group was used as the test set. This validation was repeated three times, and we took the average accuracy as the evaluation of the model.

#### Comparison With Co-Occurrence Analysis

We conducted a co-occurrence analysis (Callon et al., 1986) on the summaries and performed clinical verification on this result, which was compared with ADR discovery based on TBKG. The basic principle of co-occurrence analysis is to reflect the correlation strength between keywords by counting the co-occurrence of word pairs or noun phrases in the literature. According to this principle, we conducted frequency statistics and sorted the word pairs for all entities. Through clinical verification, we can compare the difference between the two results.

### Clinical Validation

Clinical validation was performed to validate the efficacy of our model. Osimertinib is a third-generation epidermal growth factor receptor tyrosine kinase inhibitor that is used to treat non-small-cell lung carcinomas with specific mutations (Odogwu et al., 2018). This medication was approved as an antitumor treatment in 2017 by both the Food and Drug Administration and the European Commission. As a novel antitumor drug, neither clinical trials nor real-world studies had enough data to provide an early warning of ADRs after marketing. Thus, we chose osimertinib as an example.

First, the results of our model was compared with the reported ADRs from the official manual (Supplementary Table 2) and EGFR-TKI ADR Management Chinese Expert Consensus (Anti-Cancer Association, 2019). The ADRs of osimertinib were calculated for different literature quantities from 10,667 literature abstracts on “cancer therapy” since osimertinib appears in the literature in 2014. The ADRs ranked in the top 5% according to our model were defined as important ADRs, and those ranked in the bottom 5% were defined as unlikely ADRs. Kappa index, sensitivity and specificity were used to determine the reliability of our model with the official manual of osimertinib. The difference in ADR discovery was evaluated among different literature quantities, and the difference between our model and co-occurrence analysis was also measured. All analyses were performed using SPSS (version 23.0) statistical software.

Second, we also compared the results of our model with the reported ADRs of all clinical cases from the 3rd Xiangya Hospital. The clinical data were retrospectively extracted from the structured hospital information system (HIS) of the 3rd Xiangya Hospital, Central South University (Changsha, China), which provides patient health record information, e.g., information regarding the Enterprise Master Patient Index (EMPI), laboratory tests, International Classification of Disease (ICD-10) clinical diagnosis, medical records and so on. All patients treated with osimertinib in our hospital (*n* = 8) from May 2017 to September 2020 were included in this study. ADRs (Edwards and Aronson, 2000) refer to adverse medical events that occur after a patient receives a drug but that do not necessarily have a causal relationship with the experimental drug. ADRs that meet the definition of Common Terminology Criteria for Adverse Events v4.0 (CTCAE) (US Department of Health Human Services, 2009) include the following: (1) existing exacerbations of chronic or intermittent diseases, including increased frequency and/or increased disease severity; (2) new diseases detected or diagnosed after the administration of the experimental drug, although they may have existed before the study began; (3) signs, symptoms or clinical sequelae due to suspected drug interactions; and (4) signs, symptoms, or clinical sequelae resulting from suspected overdose of an experimental drug or combination of drugs (overdose itself is not reported as an adverse event/serious adverse event). All ADRs of osimertinib during hospitalization were recorded. The Institutional Review Board of the 3rd Xiangya Hospital approved this study (No. 2020-S662).

In addition, the assumption in our study is that drugs have effects on some biomarkers and that these biomarkers are associated with the specific ADRs. And we applied TBKG to discover the adverse reactions of osimertinib and tried to find relative biomarkers that link the drugs with ADRs.

## Results

**Figure 4:**
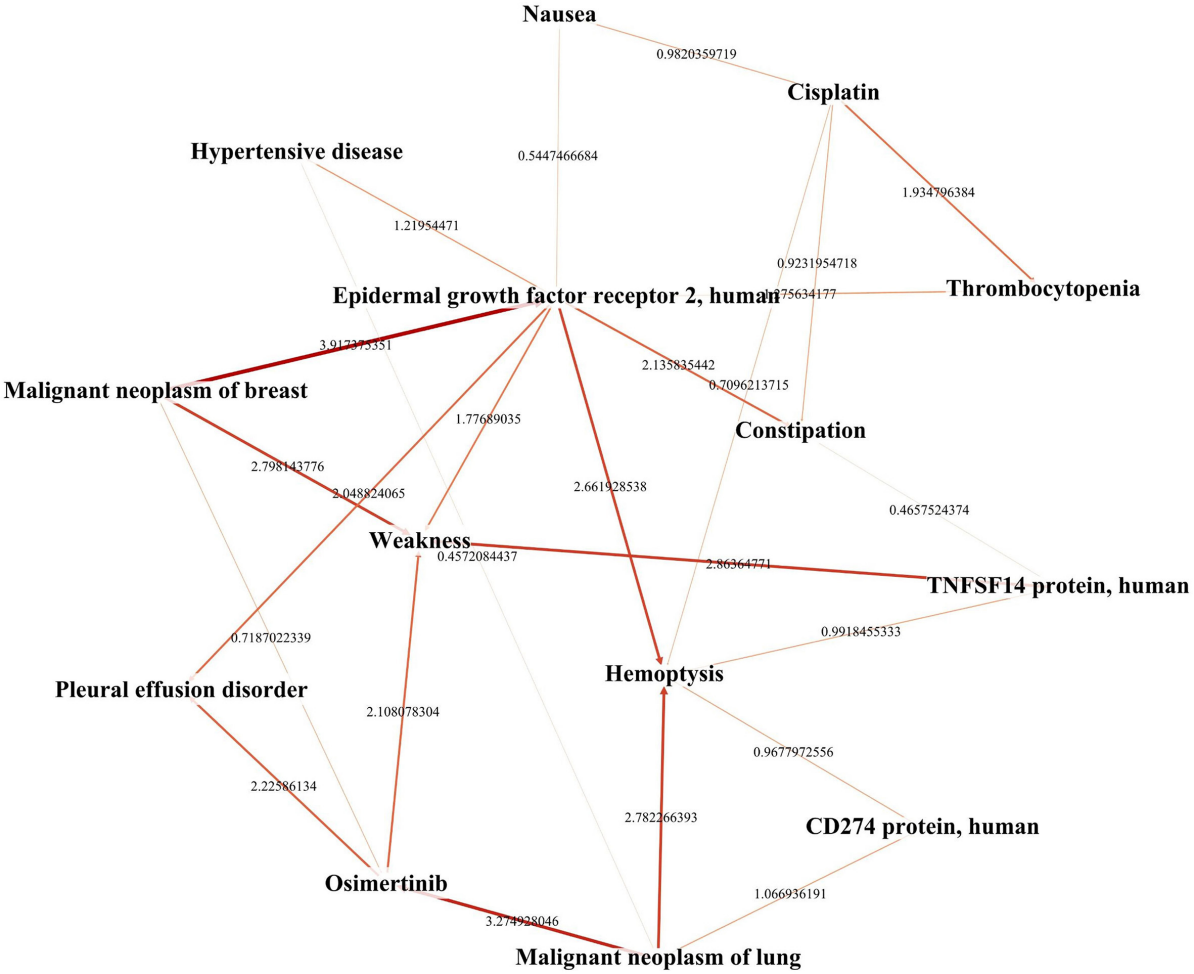
A portion of the network extracted from TBKG.

We constructed the TBKG, which is a weighted heterogeneous graph with four types of objects extracted from the MEDLINE corpus: tumor, biomarker, drug, and ADR. Six relationships were built between them (Table 2). Then, the naive Bayes model was used to determine whether relationship exist. We show a part of the TBKG in Figure 4.

**Table 2:** Numbers of entities and relationships in TBKG.

**Relationships**	**Entity numbers**	**Edge numbers**
Tumor-Biomarker	1,179–2,550	30,065
Tumor-Drug	1,179–1,806	21,293
Tumor-ADR	1,179–756	8,913
Drug-Biomarker	1,806–2,550	46,052
Drug-ADR	1,806–756	13,653
Biomarker-ADR	2,550–756	19,278

We use the biomarkers and ADRs related to the drug “osimertinib” as an example to show the TBKG results. The correlation between “osimertinib” and one of its ADRs, “Nephrosclerosis” is 4.31. The correlation result means that in the case of “osimertinib” appearing, the probability of “Nephrosclerosis” is 10.4%, while in the case of “osimertinib” not appearing, the probability of “Nephrosclerosis” is 0.5%. The greater the correlation, the more likely it is that “osimertinib” but no other factors will cause ADRs.

**Figure 5:**
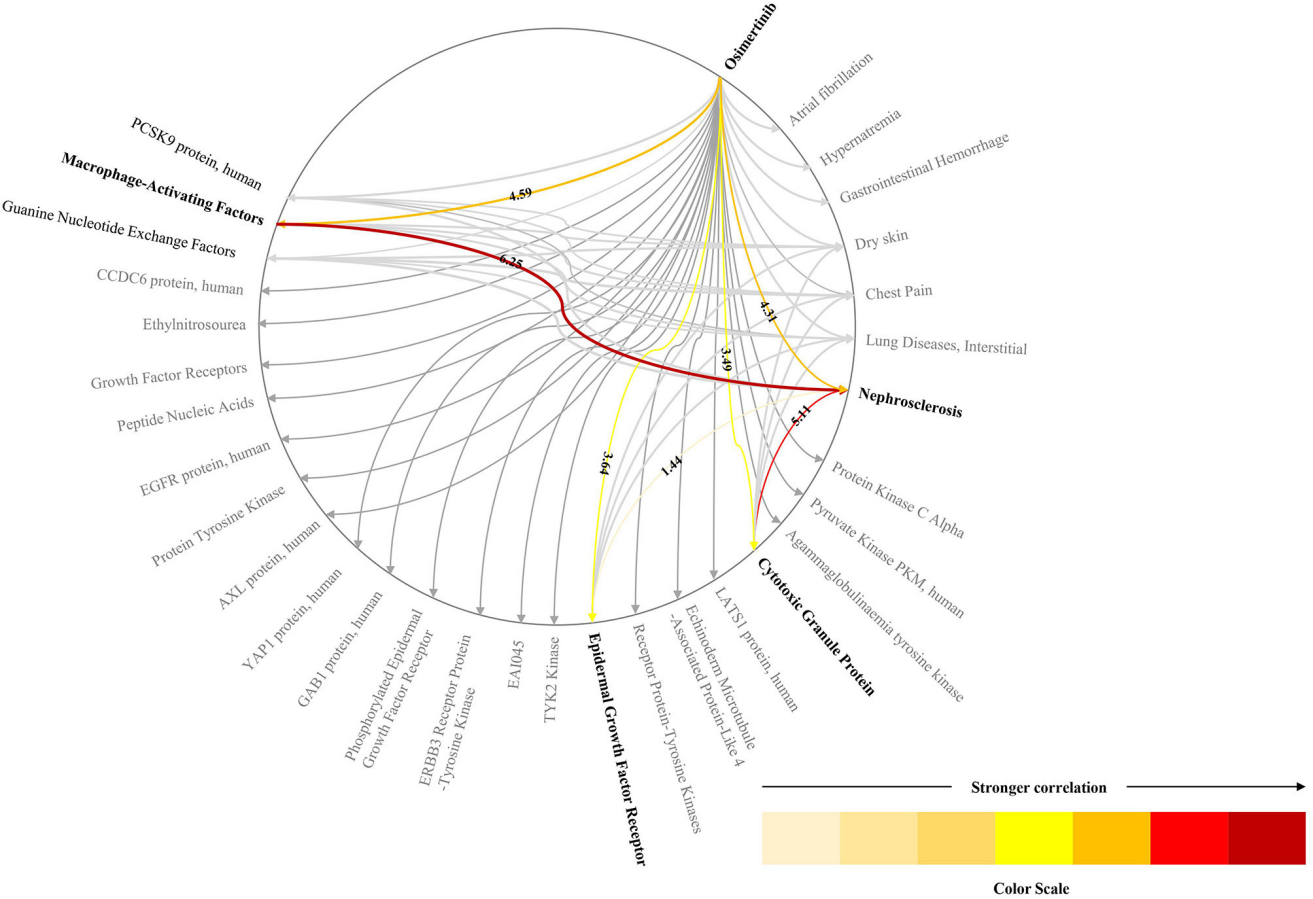
The explainable pathway between osimertinib and one of its ADRs. This figure shows the pathways between osimerinib, top 1% related biomarkers and parts of ADRs. We only highlights the pathways between osimerinib, nephrosclerosis and three biomarkers: “Cytotoxic Granule Protein,” “Epidermal Growth Factor Receptor,” and “Macrophage-Activating Factors”.

Biomarkers which link the drugs with ADRs were found in TBKG. As shown in Figure 5, the correlations between “osimertinib” and the biomarkers “Cytotoxic Granule Protein,” “Epidermal Growth Factor Receptor,” and “Macrophage-Activating Factors” are 3.49, 3.64, and 4.59, respectively. The corresponding correlations between these three tumor factors and the adverse reaction “Nephrosclerosis” are 5.11, 1.44, and 6.25. Compared with epidermal growth factor receptor and cytotoxic granule protein, macrophage activating factors seems to mediate the incident of osimertinib induced nephrosclerosis.

**Figure 6:**
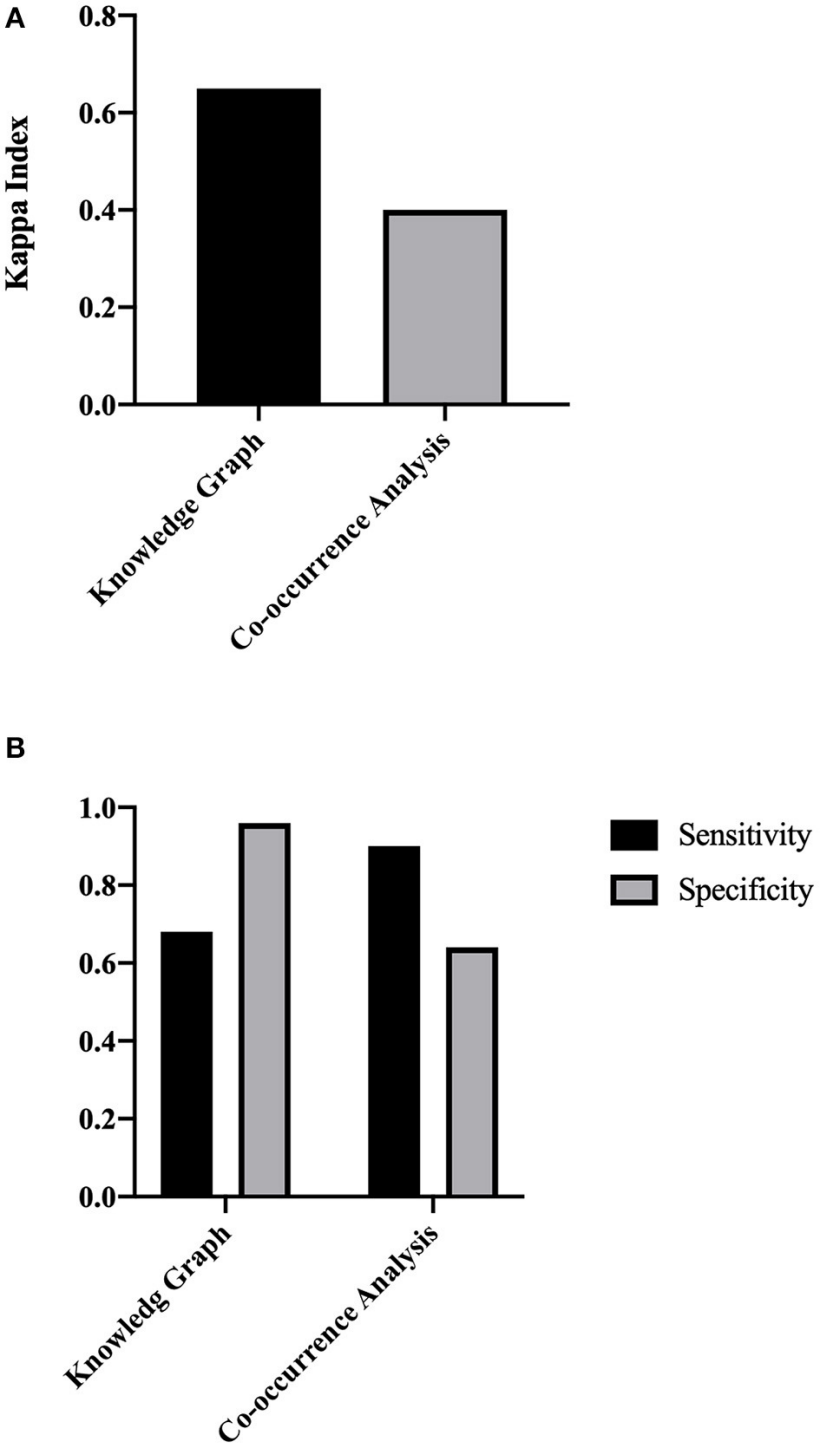
The concordance evaluation of the ADR discovery based on TBKG. **(A)** The Kappa index of TBKG compared with the co-occurrence analysis. **(B)** The sensitivity and specificity of TBKG compared with the co-occurrence analysis.

According to the calculations of our model, 775 ADRs were included in the current study, and the most important ADRs for osimertinib were ordered as follows: dry skin, paronychia inflammation, visual field defects, interstitial lung diseases, and so on. Supplementary Table 3 lists the calculation results. Our model had moderate consistency with the reports in the official manual (Kappa = 0.68, Figure 6), and better than co-occurrence (Kappa = 0.4). And compared with co-occurrence, our model had better specificity than sensitivity.

**Figure 7:**
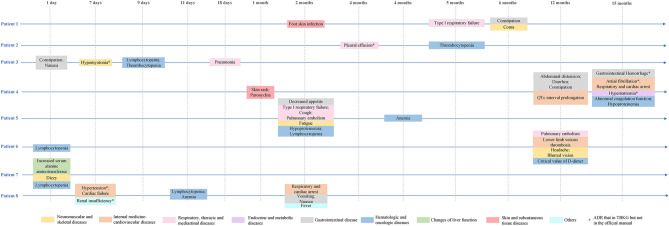
Adverse drug reactions of clinical patients receiving osimertinib. *Not mentioned in the official manual of osimertinib, but mentioned in TBKG.

Furthermore, our model could find rare and serious ADRs that had not been reported in the official manual. Eight lung adenocarcinoma patients had received osimertinib treatment in our hospital since 2017. The characteristics of the included patients are shown in Table 3. The mean age of the total population was 61 years, and 50% of the patients were female. The median follow-up time was 6 months. The most common adverse reactions in the clinical cases were lymphocytopenia (3/8), anemia (3/8), and constipation (3/8). From Figure 7, it is worth noting that some serious ADRs that has never been reported before and could be calculated by our model, for example, patient 8 developed renal failure and needed dialysis 1 week after taking osimertinib.

**Table 3:** Clinical characteristics of patients who received osimertinib at the 3rd Xiangya Hospital.

	**Patient 1**	**Patient 2**	**Patient 3**	**Patient 4**	**Patient 5**	**Patient 6**	**Patient 7**	**Patient 8**
Age at osimertinib treatment (years)	73	49	57	71	48	56	81	55
Sex	Male	Female	Male	Female	Male	Male	Female	Female
Diagnosis	Adenocarcinoma of right lung	Bronchial adenocarcinoma	Adenocarcinoma of right lung	Adenocarcinoma of left lung	Adenocarcinoma of right lung	Lung cancer^*^	Lung cancer^*^	Lung cancer^*^
Duration of cancer history	1 month	4 months	12 months	12 months	20 months	1 months	10 months	8 months
Complications	Benign prostatic hyperplasia	/	COPD	Hypertension	CKD; hypertension	/	Postoperative colon cancer; gallstone; remote cerebral infarction	/
Drug combination	Tiotropium bromide, tamsulosin, finasteride	/	Morphine, ampeptide elemente	Tramadol, celecoxib	Ulbenemax, mosapride, calcium malate, montelukast	/	Mecobalamin, trimetazidine, magnesium potassium aspartate, atorvastatin, aspirin	/
Relative gene	Undetected	EGFR (–)	EGFR (+)	EGFR (+)	EGFR (+)	Undetected	EGFR (+)	EGFR (–)
Metastasis site	Adrenal gland; bone; mediastinal lymph nodes; pleura; right subclavian lymph nodes	Lung; pleura	Mediastinal lymph nodes; right subclavian lymph nodes	Bone; liver	Lymph nodes; pericardium; pleura; enterocoelia	Bone; lung	No metastasis	Adrenal gland; bone; brain
Relative gene	Undetected	EGFR (–)	EGFR (+)	EGFR (+)	EGFR (+)	Undetected	EGFR (+)	EGFR (–)
Start date of osimertinib treatment	2016/12/28	2019/1/29	2019/12/10	2019/6/20	2019/10/28	2020/4/15	2020/4/22	2019/10/13
Follow-up time	5 months	6 months	2 months	14 months	7 months	1 months	5 months	7 months

* *= Without pathological diagnosis; / = Not mentioned in the electronic health records*.

## Discussion

Our study opens up a new direction for ADR discovery that combines the following features.

- This is the first knowledge graph-based approach to discover potential adverse reactions of antitumor drugs. By exploring the relations among tumors, biomarkers, and drugs in the knowledge graph, our approach is able to provide explanations for the potential of supervised machine learning methods.
- We contribute a dataset to study knowledge graphs for ADRs by entity extraction and relation building. We verified the efficacy of this approach with clinical data and released the data and the codes that might be valuable to the community working on emerging fields of biomedical literature mining.

Multiple scientific disciplines have been addressing the ADRs discovery problems from different perspectives (Tan et al., 2016). Not only clinical trials before marketing and reports of adverse reactions after marketing, but the detection of metabolic enzyme-related genes has also been used to discover ADRs with the development of pharmacogenomics (Phillips et al., 2001). In the data mining area (Rastegar-Mojarad et al., 2016; Santiso et al., 2018; Shen et al., 2018; Zhang et al., 2019), leveraged the existing information of the drugs and ADRs as the input for a machine learning classifier (e.g., logistic regression, decision tree, and support vector machine), which outputs a binary prediction. Recently, several methods have employed deep learning approaches (Fan et al., 2020) to detect possible ADRs with an effective integration of heterogeneous and multidimensional drug data sources. However, ADR discovery should not be as narrow as a simple true or false question. The reasons behind the ADR in the real world also exist in rich and variant biomedical literature.

Here, we proposed and verified a knowledge graph method based on literature data, that can calculate potential ADRs that never reported before. A knowledge graph is a data model that represents facts as nodes (e.g., drugs, diseases, tumors, side effects, and biomarkers) and relations between the nodes (e.g., drug-biomarker relations). Graph representations are not only able to reveal how individual semantic entities are related to each other but also appealing for human conceptual understanding. This graph structure opens up a new approach to model the abundance of ADR-related information and introduces structural information to determine whether an ADR exists for a tumor drug. It's proved to be feasible that knowledge source extraction and knowledge discovery (Rotmensch et al., 2017; Li et al., 2019; Malas et al., 2019) (e.g., drug prioritization, drug interaction, rare disease classification) by constructing health knowledge graphs. And the experimental results in this study showed that the naive Bayesian model combined building the knowledge graph could outperformed the co-occurrence analysis. Moreover, similar to a few of studies (Guney et al., 2016; Bean et al., 2017), we calculated the potential ADRs by measuring the distance in the graph between the drug, biomarkers, diseases and ADRs (i.e., the drug that cures a disease that are associated with an ADR).The underlying assumption was that a short distance between drugs and ADRs meant that the drug was likely to cause the ADRs.

Our findings not only uncover simple ADRs but also provide explanations, i.e., the paths of “tumor-biomarker-drug” in the knowledge graph. Capturing detailed ADR information may help to obtain in-depth insights into the underlying mechanisms. Previous studies focused on the integration of drug structure or chemical features (Frid and Matthews, 2010; Pauwels et al., 2011) or gene expression (Wang et al., 2016) or drug target (Párez-Nueno et al., 2015) to optimize ADR prediction models. Risk factors of drug-induced organ damage include drug overdose, drug-drug interactions and drug-related adverse effects, and the discovery of the early biomarkers and development of accurate diagnostic methods are effective prevention strategies for organ damage (Wu and Huang, 2018).

Few studies focus on molecular mechanisms' interpretability in ADR discovery (Hristovski et al., 2016). The spread through relevance in the knowledge graph provides convenience for interpretability. The assumption in our study is that drugs have effects on some biomarkers (proteins, enzymes, and so on) and that these biomarkers are associated with the specific ADRs. Therefore, we try to find biomarkers link the drugs with ADRs in TBKG. The quality of the explanations for the ADRs provided in our approach largely depends on the precision of this knowledge graph, which have been validated whether from the model performance or clinical perspective in this study. Thus, our method can offer better understanding of the biomarkers of ADRs, which could not only significantly predict the potential ADRs before the drug development, but can also provide oncologists with the opportunity to quickly predict patients' sensitivity to the ADRs.

Although important discoveries have been revealed by the current study, there are also limitations. First, the calculated drug-biomarker combinations cannot distinguish between a drug-treatment relationship, a drug-ADR relationship, or a “is not a target drug” type of relationship. However, this is one of major shifts of the big data mindset–a growing emphasis on correlations rather than a continuing quest for elusive causality. In traditional clinical trials, both causal investigations and correlation analysis begin with a hypothesis that is tested to be falsified or verified with little data available. In the age of big data, this type of noncausal analysis will help us understand the “what” rather than the “why.” Nonetheless, the construction of relation extraction templates based on the domain knowledge graph (e.g., increasing risk, causing) is encouraging. Second, this study lacks the attention to drug-drug interaction. The use of antitumor drugs often results in the use of other agents to reduce or prevent ADRs and cancer itself increases the need for more medications, which could increase the risk of ADRs. Third, this study focused on ADR discovery based on medical literature. Although compared with the detection of ADRs using clinical data alone, the ADR discovery based on literature have the potential to find the unreported ADR as in our study. For future research, we will improve this knowledge graph-based approach by data fusion and knowledge representation. Finally, these types of recommendations should be assessed by studying the following questions: How many clinicians read them? Are they applied? How effective have they been in reducing the incidence of the complications of hypertension and adverse drug effects? Further prospective clinical trials evaluating the effectiveness of this type of decision support will be explored as the next steps.

## Conclusion

In summary, the approach described a reliable method for ADR discovery of antitumor drugs and provided explanations of predicted ADRs by exploring the relations among tumors, biomarkers, and drugs in the knowledge graph. This study contributes a dataset to study knowledge graphs for ADRs by entity extraction and relation building; and releases the data and the codes that might be valuable to the community working biomedical literature mining. These findings also provide impetus for the mechanism research of ADRs and therefore offer biomarkers to predict ADRs.

## Data Availability Statement

The original contributions presented in the study are included in the article/Supplementary Materials, further inquiries can be directed to the corresponding author/s.

## Ethics Statement

The studies involving human participants were reviewed and approved by The IRB of Thira Xiangya Hospital, Central South University. The ethics committee waived the requirement of written informed consent for participation.

## Author Contributions

HT, TL, WO, LG, YT, XH, and WH collected and analysis the clinical data. XL, MW, HW, JS, and XM conceived and designed the experiments, performed the experiments, and wrote the paper. All authors contributed to the conceptual design of the study, the data collection planning and the initial drafting of the manuscript.

## Conflict of Interest

The authors declare that the research was conducted in the absence of any commercial or financial relationships that could be construed as a potential conflict of interest.
