# Supplementary material for: Adverse Drug Reaction Discovery Using a Tumor-Biomarker Knowledge Graph
Source: Front Genet. 2021 Jan 12;11:625659. doi: 10.3389/fgene.2020.625659 (PMC7873847; doi:10.3389/fgene.2020.625659)
Supplement: Supplementary file 1 [file Table_1.DOCX]

**Supplementary Table 1. The source vocabulary used to build the dictionary**

| Vocabulary | Abbreviation | Purpose |
| --- | --- | --- |
| Alcohol and Other Drug Thesaurus | AOD | AOD serves as a conceptual framework for researchers and practitioners in the alcohol and other drug field, and as a controlled vocabulary for indexing and information retrieval in database systems. |
| CRISP Thesaurus | CSP | CRISP is a terminology used for indexing biomedical information. |
| MeSH | MSH | The MeSH thesaurus is a controlled vocabulary used for indexing, cataloging, and searching for biomedical and health-related information and documents. It enables retrieval systems, such as PubMed, to provide subject searching of data. |
| NCI Thesaurus | NCI | The NCI Thesaurus is a reference terminology and biomedical ontology. |
| RXNORM | RXNORM | The goal of RxNorm is to allow various systems using different drug nomenclatures to share data efficiently. RxNorm provides standard names for clinical drugs (active ingredient + strength + dose form) and for dose forms as administered to a patient. It provides links from clinical drugs, both branded and generic, to their active ingredients, drug components (active ingredient + strength), and related brand names. |
| SNOMED CT, US Edition | SNOMEDCT_US | SNOMED CT aims to improve patient care through the development of systems to record health care encounters accurately. The US Edition of SNOMED CT facilitates the use of SNOMED CT as a primary coding terminology for clinical information in electronic health records, research and clinical trials. It is used with RxNorm and LOINC to cover medications and laboratory tests needs in the US. |
| WHOART | WHO | WHO-ART is used for coding clinical information related to adverse drug reactions. |
