# Supplementary material for: Adverse Drug Reaction Discovery Using a Tumor-Biomarker Knowledge Graph
Source: Front Genet. 2021 Jan 12;11:625659. doi: 10.3389/fgene.2020.625659 (PMC7873847; doi:10.3389/fgene.2020.625659)
Supplement: Supplementary file 2 [file Table_2.DOCX]

**Supplementary Table 2. Adverse Reactions of Osimertinib reported by the official manual**

| MedDRA SOC | MedDRA |
| --- | --- |
| Respiratory, thoracic and mediastinal diseases | Cough^1^ |
|  | Dyspnea^1^ |
|  | Upper respiratory tract infection^1^ |
|  | Interstitial lung disease^2^ |
|  | Pneumonia^2^ |
|  | Pulmonary embolism^2^ |
| Gastrointestinal disease | Diarrhea^1^ |
|  | Stomatitis^1^ |
|  | Decreased appetite^1^ |
|  | Constipation^1^ |
|  | Nausea^1^ |
|  | Vomiting^1^ |
| Changes of liver function | Increased serum aspartate aminotransferase^1a^ |
|  | Increased serum alanine aminotransferase^1a^ |
|  | Hyperbilirubinemia^1^ |
| Internal medicine-cardiovascular diseases | Cardiovascular: Prolonged QT interval on ECG^2b^ |
|  | Decreased left ventricular ejection fraction^2^ |
|  | Myocarditis^4^ |
| Neuromuscular and skeletal diseases | Fatigue^1^ |
|  | Headache^1^ |
|  | Asthenia^4^ |
| Endocrine and metabolic diseases | Hypermagnesemia^1a^ |
|  | Hypokalemia^1a^ |
|  | Hyponatremia^3a^ |
| Hematologic and oncologic diseases | Lymphocytopenia^1a^ |
|  | Anemia^1a^ |
|  | Thrombocytopenia^1a^ |
|  | Neutropenia^1a^ |
| Skin and subcutaneous tissue diseases | Skin rash^1c^ |
|  | Nail disease^1d^ |
|  | Xeroderma^1e^ |
|  | Pruritus^1f^ |
| Ophthalmic disease | Keratitis^3g^ |
|  | Eyelid pruritus^4^ |
| Others | Fever^1^ |

MedDRA: International Technical Coordination Committee for the Registration of drugs for Human use（ICH）International Dictionary of Medical terms; SOA: Systematic organ classification;

^1^Very common (the incidence≥10%) according to the reports of the official manual; ^2^Common (1%≤the incidence＜10%); ^3^Occasional(0.1%≤the incidence＜1%); ^4^Unspecified(It is impossible to elaborate on the available data);

^a^Indicates that the incidence of laboratory tests is not the reported incidence of adverse events;

^b^It indicates the incidence of prolonged QTcF > 500 msec in patients;

^c^Reported cases that include the following classification terms for rash events: Rash, systemic rash, red rash, macular rash, macular papules, papules, pustules, itching rash, vesicular rash, follicular rash, erythema, folliculitis, acne, dermatitis, acne-like dermatitis, drug eruption and skin erosion;

^d^Reported cases including the following classified terms: nail bed disease, nail bed inflammation, nail bed infection, fingernail discoloration, fingernail pigmentation, fingernail disease, fingernail toxicity, fingernail dystrophy, fingernail infection, fingernail sheer, brittle nail, nail peeling, nail softening, onychomyelitis;

^e^Reported cases including the following classified terms: dry skin, cracked skin, xerosis, eczema, xeroderma;

^f^Reported cases including the following categorized terms: itching, systemic itching;

^g^Reported cases including the following classified terms: keratitis, punctate keratitis, corneal erosion, corneal epithelial defect.
