# Supplementary material for: Adverse Drug Reaction Discovery Using a Tumor-Biomarker Knowledge Graph
Source: Front Genet. 2021 Jan 12;11:625659. doi: 10.3389/fgene.2020.625659 (PMC7873847; doi:10.3389/fgene.2020.625659)
Supplement: Supplementary file 3 [file Table_3.DOCX]

**Supplementary Table 3. Lists of ADRs of Osimertinib calculated by the TBKG**

| **Ranking Order** | **ADR** | **Correlation coefficient** |
| --- | --- | --- |
| 1 | Dry skin | 4.053988 |
| 2 | Paronychia Inflammation | 4.005198 |
| 3 | Visual field defects | 3.899838 |
| 4 | Lung Diseases, Interstitial | 3.622206 |
| 5 | Interstitial lung fibrosis | 3.494373 |
| 6 | Erythema Multiforme | 3.494373 |
| 7 | Pneumoconiosis | 3.494373 |
| 8 | Keratopathy | 3.494373 |
| 9 | Corneal Ulcer | 3.494373 |
| 10 | Urticaria | 3.494373 |
| 11 | Hemolytic-Uremic Syndrome | 3.494373 |
| 12 | Eosinophilic Pneumonia | 3.494373 |
| 13 | Femoral Fractures | 3.494373 |
| 14 | Drug-induced hyperpyrexia | 3.494373 |
| 15 | Pulmonary Eosinophilia | 3.494373 |
| 16 | Bronchiectasis | 3.494373 |
| 17 | Hyperreflexia | 3.494373 |
| 18 | Prolonged QT interval | 3.494373 |
| 19 | Alveolitis | 3.494373 |
| 20 | Intracranial Hypertension | 3.494373 |
| 21 | Decrease in appetite | 2.983547 |
| 22 | Alanine Aminotransferase Increased | 2.801225 |
| 23 | Lymphocytosis | 2.801225 |
| 24 | Pneumonia, Interstitial | 2.801225 |
| 25 | Oral Ulcer | 2.801225 |
| 26 | Hypopituitarism | 2.801225 |
| 27 | Blurred vision | 2.801225 |
| 28 | Aspartate Aminotransferase Increased | 2.801225 |
| 29 | Meningitis | 2.801225 |
| 30 | Hypesthesia | 2.801225 |
| 31 | Torsades de Pointes | 2.801225 |
| 32 | Abnormal vision | 2.801225 |
| 33 | Xerosis | 2.801225 |
| 34 | Exanthema | 2.486144 |
| 35 | Uveitis | 2.39576 |
| 36 | Acne | 2.39576 |
| 37 | Rectal hemorrhage | 2.39576 |
| 38 | nervous system disorder | 2.39576 |
| 39 | Retinal Diseases | 2.39576 |
| 40 | Sudden death | 2.39576 |
| 41 | Hepatotoxicity | 2.331222 |
| 42 | Chest Pain | 2.2904 |
| 43 | Pleural effusion disorder | 2.225861 |
| 44 | Coughing | 2.172617 |
| 45 | Ulcer on tongue | 2.108078 |
| 46 | Syncope | 2.108078 |
| 47 | Pelvic Pain | 2.108078 |
| 48 | Rhabdomyolysis | 2.108078 |
| 49 | Varicosity | 2.108078 |
| 50 | Esophageal Varices | 2.108078 |
| 51 | Slow shallow breathing | 2.108078 |
| 52 | Rhinitis | 2.108078 |
| 53 | Bradycardia | 2.108078 |
| 54 | Aplastic Anemia | 2.108078 |
| 55 | Trigeminal Neuralgia | 2.108078 |
| 56 | Pain in lower limb | 2.108078 |
| 57 | Disorder of tendon | 2.108078 |
| 58 | Fasciitis, Plantar | 2.108078 |
| 59 | Low Birth Weights | 2.108078 |
| 60 | Dermoid Cyst | 2.108078 |
| 61 | Hemiplegia | 2.108078 |
| 62 | Endocarditis | 2.108078 |
| 63 | Hemorrhoids | 2.108078 |
| 64 | Intracranial Hemorrhage | 2.108078 |
| 65 | Enteritis | 2.108078 |
| 66 | Peripheral Ischemia | 2.108078 |
| 67 | Nail changes | 2.108078 |
| 68 | Monocytosis | 2.108078 |
| 69 | Septicemia | 2.108078 |
| 70 | Upper Respiratory Infections | 2.108078 |
| 71 | Pleurisy | 2.108078 |
| 72 | Anaphylactic shock | 2.108078 |
| 73 | anaphylaxis | 2.108078 |
| 74 | Necrotizing fasciitis | 2.108078 |
| 75 | Fulminant Hepatitis | 2.108078 |
| 76 | Mucosal ulcer | 2.108078 |
| 77 | Stevens-Johnson Syndrome | 2.108078 |
| 78 | Subarachnoid Hemorrhage | 2.108078 |
| 79 | Urethritis | 2.108078 |
| 80 | Disease Exacerbation | 2.108078 |
| 81 | Hepatomegaly | 2.108078 |
| 82 | Abdominal discomfort | 2.108078 |
| 83 | Hemiparesis | 2.108078 |
| 84 | Vision Disorders | 2.108078 |
| 85 | Increased bilirubin level (finding) | 2.108078 |
| 86 | Dermatitis, Atopic | 2.108078 |
| 87 | Cellulitis | 2.108078 |
| 88 | Chest discomfort | 2.108078 |
| 89 | Thyroiditis | 2.108078 |
| 90 | Granulocytopenic disorder | 2.108078 |
| 91 | Hemoglobin increased | 2.108078 |
| 92 | Idiopathic Pulmonary Fibrosis | 2.108078 |
| 93 | Testicular Diseases | 2.108078 |
| 94 | Unspecified Abortion | 2.108078 |
| 95 | Pituitary Neoplasms | 2.108078 |
| 96 | Increased frequency of micturition | 2.108078 |
| 97 | Tenosynovitis | 2.108078 |
| 98 | Spasm | 2.108078 |
| 99 | Bronchospasm | 2.108078 |
| 100 | Muscle Weakness | 2.108078 |
| 101 | Absent reflex | 2.108078 |
| 102 | Osteoma | 2.108078 |
| 103 | Hydrocephalus | 2.108078 |
| 104 | Dermatitis acneiform | 2.108078 |
| 105 | Obstructive Hydrocephalus | 2.108078 |
| 106 | Intolerant of heat | 2.108078 |
| 107 | Retching | 2.108078 |
| 108 | Hypovolemia | 2.108078 |
| 109 | Cerebellar hemorrhage | 2.108078 |
| 110 | Pure Red-Cell Aplasia | 2.108078 |
| 111 | Voice Disturbance | 2.108078 |
| 112 | Petechiae | 2.108078 |
| 113 | Carcinoma in situ of uterine cervix | 2.108078 |
| 114 | Melanocytic nevus | 2.108078 |
| 115 | Nevus | 2.108078 |
| 116 | Hemianopsia | 2.108078 |
| 117 | Atherosclerosis | 2.108078 |
| 118 | Synovitis | 2.108078 |
| 119 | Acneiform Eruptions | 2.108078 |
| 120 | Pulmonary Emphysema | 2.108078 |
| 121 | Muscle hypertrophy | 2.108078 |
| 122 | Nephrosis | 2.108078 |
| 123 | Lymphangioma | 2.108078 |
| 124 | Hypoproteinemia | 2.108078 |
| 125 | Tendinitis | 2.108078 |
| 126 | Drug Eruptions | 2.108078 |
| 127 | Herpes zoster disease | 2.108078 |
| 128 | Hypernatremia | 2.108078 |
| 129 | Bursitis | 2.108078 |
| 130 | Pemphigus | 2.108078 |
| 131 | HIV Seropositivity | 2.108078 |
| 132 | Hypertriglyceridemia | 2.108078 |
| 133 | Intraabdominal hemorrhage | 2.108078 |
| 134 | Ovarian Cysts | 2.108078 |
| 135 | Hypochondriasis | 2.108078 |
| 136 | Asphyxia | 2.108078 |
| 137 | Cholecystolithiasis | 2.108078 |
| 138 | Bowel sounds | 2.108078 |
| 139 | Temporomandibular Joint Dysfunction Syndrome | 2.108078 |
| 140 | Hyperthyroidism | 2.108078 |
| 141 | Ovarian hyperstimulation | 2.108078 |
| 142 | Leg edema | 2.108078 |
| 143 | Sarcoidosis | 2.108078 |
| 144 | Stomach ache | 2.108078 |
| 145 | Staggering gait | 2.108078 |
| 146 | Tracheoesophageal Fistula | 2.108078 |
| 147 | Bladder neck obstruction | 2.108078 |
| 148 | Iron deficiency anemia | 2.108078 |
| 149 | Malabsorption | 2.108078 |
| 150 | Angina Pectoris | 2.108078 |
| 151 | Paroxysmal atrial fibrillation | 2.108078 |
| 152 | Polydipsia | 2.108078 |
| 153 | Panniculitis | 2.108078 |
| 154 | Lobular panniculitis | 2.108078 |
| 155 | Abnormal pigmentation | 2.108078 |
| 156 | Burning sensation of skin | 2.108078 |
| 157 | Pancreatitis, Chronic | 2.108078 |
| 158 | Eosinophilia | 2.108078 |
| 159 | Raised TSH level | 2.108078 |
| 160 | Angioma | 2.108078 |
| 161 | Peripheral Vascular Diseases | 2.108078 |
| 162 | Down Syndrome | 2.108078 |
| 163 | Pelvic Inflammatory Disease | 2.108078 |
| 164 | Ventricular arrhythmia | 2.108078 |
| 165 | Benign digestive system neoplasms | 2.108078 |
| 166 | Esophageal Stricture | 2.108078 |
| 167 | Irritable Mood | 2.108078 |
| 168 | Chest tightness | 2.108078 |
| 169 | Hepatic Veno-Occlusive Disease | 2.108078 |
| 170 | Fecal Incontinence | 2.108078 |
| 171 | Palpitations | 2.108078 |
| 172 | Granuloma | 2.108078 |
| 173 | Lymphangitis | 2.108078 |
| 174 | Erythrocyte sedimentation rate raised | 2.108078 |
| 175 | Facial edema | 2.108078 |
| 176 | Turner Syndrome | 2.108078 |
| 177 | Pallor | 2.108078 |
| 178 | Laryngeal Edema | 2.108078 |
| 179 | Loss of motivation | 2.108078 |
| 180 | Klinefelter Syndrome | 2.108078 |
| 181 | Vaginal Discharge | 2.108078 |
| 182 | Epilepsy, Temporal Lobe | 2.108078 |
| 183 | Cerebral Ischemia | 2.108078 |
| 184 | Vascular insufficiency of intestine | 2.108078 |
| 185 | Gastric hemorrhage | 2.108078 |
| 186 | Bleeding gastric ulcer | 2.108078 |
| 187 | Torticollis | 2.108078 |
| 188 | Increased number of platelets | 2.108078 |
| 189 | Shock, Cardiogenic | 2.108078 |
| 190 | Emotional problems | 2.108078 |
| 191 | Scleroderma | 2.108078 |
| 192 | Urogenital Abnormalities | 2.108078 |
| 193 | Dysmenorrhea | 2.108078 |
| 194 | Sunburn | 2.108078 |
| 195 | Uterine Neoplasms | 2.108078 |
| 196 | Melanosis | 2.108078 |
| 197 | Large for gestational age | 2.108078 |
| 198 | Decreased Libido | 2.108078 |
| 199 | Ulcerative Colitis | 2.108078 |
| 200 | Unplanned pregnancy | 2.108078 |
| 201 | Congestive heart failure | 2.108078 |
| 202 | Infection of ear | 2.108078 |
| 203 | Otitis Media | 2.108078 |
| 204 | Scoliosis, unspecified | 2.108078 |
| 205 | Burning feeling vagina | 2.108078 |
| 206 | Stridor | 2.108078 |
| 207 | Amyloidosis | 2.108078 |
| 208 | Hypophosphatemia | 2.108078 |
| 209 | Secondary glaucoma | 2.108078 |
| 210 | Unconscious State | 2.108078 |
| 211 | Hepatitis, Toxic | 2.108078 |
| 212 | Lower abdominal pain | 2.108078 |
| 213 | Excessive tearing | 2.108078 |
| 214 | Renal tubular necrosis | 2.108078 |
| 215 | Acute Kidney Tubular Necrosis | 2.108078 |
| 216 | Psychomotor Agitation | 2.108078 |
| 217 | Hallucinations | 2.108078 |
| 218 | Hemometra | 2.108078 |
| 219 | Myoclonus | 2.108078 |
| 220 | Tremor | 2.108078 |
| 221 | Myelitis | 2.108078 |
| 222 | Autonomic nervous system disorders | 2.108078 |
| 223 | Pericarditis, Constrictive | 2.108078 |
| 224 | Liver function tests abnormal finding | 2.108078 |
| 225 | Sick Sinus Syndrome | 2.108078 |
| 226 | Thrombocytosis | 2.108078 |
| 227 | Facial Pain | 2.108078 |
| 228 | Diplopia | 2.108078 |
| 229 | Periosteal Disorder | 2.108078 |
| 230 | Fullness abdominal | 2.108078 |
| 231 | Hyperphagia | 2.108078 |
| 232 | abnormal glucose tolerance test | 2.108078 |
| 233 | Aortic Valve Stenosis | 2.108078 |
| 234 | Difficult intubation | 2.108078 |
| 235 | Keloid | 2.108078 |
| 236 | Tattoo disorder | 2.108078 |
| 237 | Respiratory arrest | 2.108078 |
| 238 | Mask-like facies | 2.108078 |
| 239 | Mydriasis | 2.108078 |
| 240 | Loin pain | 2.108078 |
| 241 | Drug withdrawal syndrome | 2.108078 |
| 242 | Hemothorax | 2.108078 |
| 243 | Gastroparesis | 2.108078 |
| 244 | nipple discharge | 2.108078 |
| 245 | Acute Erythroblastic Leukemia | 2.108078 |
| 246 | Hiccough | 2.108078 |
| 247 | Dysesthesia | 2.108078 |
| 248 | Lichen disease | 2.108078 |
| 249 | Lichen Planus | 2.108078 |
| 250 | Hyperkeratosis | 2.108078 |
| 251 | Pre-Eclampsia | 2.108078 |
| 252 | Sudden infant death syndrome | 2.108078 |
| 253 | Greasy skin | 2.108078 |
| 254 | Hyphema | 2.108078 |
| 255 | Hypogammaglobulinemia | 2.108078 |
| 256 | Mucous membrane swelling | 2.108078 |
| 257 | Antibodies, Antinuclear | 2.108078 |
| 258 | Gastric ulcer with perforation | 2.108078 |
| 259 | Vitamin D Deficiency | 2.108078 |
| 260 | Hyperalgesia | 2.108078 |
| 261 | Tachycardia | 2.108078 |
| 262 | Bulla | 2.108078 |
| 263 | Sinus Tachycardia | 2.108078 |
| 264 | Fissure in skin | 2.108078 |
| 265 | Gynecomastia | 2.108078 |
| 266 | Erythropenia | 2.108078 |
| 267 | Abdominal colic | 2.108078 |
| 268 | Vitiligo | 2.108078 |
| 269 | nervous system complication | 2.108078 |
| 270 | Cerebral atrophy | 2.108078 |
| 271 | Memory impairment | 2.108078 |
| 272 | Autistic Disorder | 2.108078 |
| 273 | Cervical lymphadenopathy | 2.108078 |
| 274 | Influenza-like symptoms | 2.108078 |
| 275 | Attention deficit hyperactivity disorder | 2.108078 |
| 276 | Spinal Cord Injuries | 2.108078 |
| 277 | Poliomyelitis | 2.108078 |
| 278 | Migraine Disorders | 2.108078 |
| 279 | Kidney Calculi | 2.108078 |
| 280 | Guillain-Barre Syndrome | 2.108078 |
| 281 | Benign neoplasm of pituitary gland | 2.108078 |
| 282 | Pruritus | 2.108078 |
| 283 | Chickenpox | 2.108078 |
| 284 | Skin Diseases, Infectious | 2.108078 |
| 285 | Vasospasm | 2.108078 |
| 286 | Perforation of stomach | 2.108078 |
| 287 | Mediastinitis | 2.108078 |
| 288 | Rubinstein-Taybi Syndrome | 2.108078 |
| 289 | Menstruation Disturbances | 2.108078 |
| 290 | Atrial Septal Defects | 2.108078 |
| 291 | Psychotic Disorders | 2.108078 |
| 292 | Hyperkalemia | 2.108078 |
| 293 | Cerebral Hemorrhage | 2.108078 |
| 294 | Dizziness | 2.108078 |
| 295 | Mood Disorders | 2.108078 |
| 296 | Nervousness | 2.073789 |
| 297 | Diarrhea | 2.012768 |
| 298 | Cardiac Arrhythmia | 1.953928 |
| 299 | Cerebral Infarction | 1.884935 |
| 300 | Granulomatosis with polyangiitis | 1.884935 |
| 301 | Lack of Efficacy | 1.721305 |
| 302 | Xeroderma | 1.702613 |
| 303 | Polycythemia | 1.702613 |
| 304 | Aphasia | 1.702613 |
| 305 | Azoospermia | 1.702613 |
| 306 | Hernia | 1.702613 |
| 307 | Atelectasis | 1.702613 |
| 308 | Multiple birth (finding) | 1.702613 |
| 309 | Ototoxicity | 1.702613 |
| 310 | Fasciitis | 1.702613 |
| 311 | Hypocalcemia | 1.702613 |
| 312 | Phlebitis | 1.702613 |
| 313 | Irritable Bowel Syndrome | 1.702613 |
| 314 | Peptic Ulcer | 1.702613 |
| 315 | Cryptorchidism | 1.702613 |
| 316 | Vascular Diseases | 1.702613 |
| 317 | Anemia, Hemolytic | 1.702613 |
| 318 | Dermatomyositis | 1.702613 |
| 319 | Medication Error | 1.702613 |
| 320 | Atypical lymphocyte | 1.702613 |
| 321 | Neuroleptic Malignant Syndrome | 1.702613 |
| 322 | Lethargy | 1.702613 |
| 323 | Pulmonary Fibrosis | 1.702613 |
| 324 | Elevated alpha-fetoprotein | 1.702613 |
| 325 | Oligospermia | 1.702613 |
| 326 | Hyperbilirubinemia | 1.702613 |
| 327 | Hypoglycemia | 1.702613 |
| 328 | Musculoskeletal Diseases | 1.702613 |
| 329 | Pancreatitis, Acute | 1.702613 |
| 330 | Muscular stiffness | 1.702613 |
| 331 | Pancytopenia | 1.702613 |
| 332 | Muscular Dystrophy | 1.702613 |
| 333 | Mastodynia | 1.702613 |
| 334 | Lupus Erythematosus, Systemic | 1.702613 |
| 335 | Malignant neoplasm of pharynx | 1.702613 |
| 336 | Retinopathy of Prematurity | 1.702613 |
| 337 | Thirst | 1.702613 |
| 338 | Pheochromocytoma | 1.702613 |
| 339 | Duodenal Ulcer | 1.702613 |
| 340 | Vasculitis | 1.702613 |
| 341 | Parkinson Disease | 1.702613 |
| 342 | Difficulty sleeping | 1.702613 |
| 343 | Tumor Lysis Syndrome | 1.702613 |
| 344 | Graft Rejection | 1.702613 |
| 345 | Fibroid Tumor | 1.702613 |
| 346 | Epididymitis | 1.702613 |
| 347 | Glomerulonephritis | 1.702613 |
| 348 | Hematemesis | 1.702613 |
| 349 | Epistaxis | 1.702613 |
| 350 | Appendicitis | 1.702613 |
| 351 | Inappropriate ADH Syndrome | 1.702613 |
| 352 | Neonatal Abstinence Syndrome | 1.702613 |
| 353 | Gastroesophageal reflux disease | 1.702613 |
| 354 | Maculopapular Lesion | 1.702613 |
| 355 | Abnormal coordination | 1.702613 |
| 356 | Arterial thrombosis | 1.702613 |
| 357 | Agitation | 1.702613 |
| 358 | Sleep Apnea Syndromes | 1.702613 |
| 359 | Chronic heart failure | 1.702613 |
| 360 | Hoarseness | 1.702613 |
| 361 | Bowen's Disease | 1.702613 |
| 362 | Paralytic Ileus | 1.702613 |
| 363 | Gall Bladder Diseases | 1.702613 |
| 364 | Pyloric Stenosis | 1.702613 |
| 365 | Hyperpigmentation | 1.702613 |
| 366 | Craniopharyngioma | 1.702613 |
| 367 | Jaundice, Obstructive | 1.702613 |
| 368 | Mood swings | 1.702613 |
| 369 | Chills | 1.702613 |
| 370 | Premenstrual Tension | 1.702613 |
| 371 | Lupus Erythematosus, Discoid | 1.702613 |
| 372 | Respiratory Distress Syndrome, Newborn | 1.702613 |
| 373 | Ataxia | 1.702613 |
| 374 | Anal pain | 1.702613 |
| 375 | Gangrene | 1.702613 |
| 376 | Congenital chromosomal disease | 1.702613 |
| 377 | Skin Ulcer | 1.702613 |
| 378 | Somnolence | 1.702613 |
| 379 | Hyperammonemia | 1.702613 |
| 380 | Necrolysis epidermal | 1.702613 |
| 381 | Spinal Fractures | 1.702613 |
| 382 | Pericarditis | 1.702613 |
| 383 | Numbness | 1.702613 |
| 384 | Surgical Wound Infection | 1.702613 |
| 385 | Rectal pain | 1.702613 |
| 386 | Pericardial effusion | 1.702613 |
| 387 | Nephritis, Interstitial | 1.702613 |
| 388 | Comatose | 1.702613 |
| 389 | Pneumonitis | 1.702613 |
| 390 | Neurologic Symptoms | 1.702613 |
| 391 | Liver Dysfunction | 1.597253 |
| 392 | Myocardial Infarction | 1.548463 |
| 393 | Pulmonary Embolism | 1.414931 |
| 394 | Pneumothorax | 1.414931 |
| 395 | Dyspnea | 1.414931 |
| 396 | Polyarteritis Nodosa | 1.414931 |
| 397 | Hypercholesterolemia | 1.414931 |
| 398 | Bone necrosis | 1.414931 |
| 399 | Coronary heart disease | 1.414931 |
| 400 | Cholecystitis | 1.414931 |
| 401 | Pathological fracture | 1.414931 |
| 402 | Wakefulness | 1.414931 |
| 403 | Small bowel obstruction | 1.414931 |
| 404 | Malignant neoplasm of male breast | 1.414931 |
| 405 | Dislocations | 1.414931 |
| 406 | Low Back Pain | 1.414931 |
| 407 | Paresthesia | 1.414931 |
| 408 | Fanconi Syndrome | 1.414931 |
| 409 | Dyspepsia | 1.414931 |
| 410 | Intestinal Perforation | 1.414931 |
| 411 | Dementia | 1.414931 |
| 412 | Detachment psychological | 1.414931 |
| 413 | Portal Hypertension | 1.414931 |
| 414 | Social disinhibition | 1.414931 |
| 415 | Glaucoma | 1.414931 |
| 416 | Leukocytosis | 1.414931 |
| 417 | Septic Shock | 1.414931 |
| 418 | Dysgeusia | 1.414931 |
| 419 | Skin necrosis | 1.414931 |
| 420 | Viral hepatitis | 1.414931 |
| 421 | Gastric ulcer | 1.414931 |
| 422 | Cholelithiasis | 1.414931 |
| 423 | Upper abdominal pain | 1.414931 |
| 424 | Rib Fractures | 1.414931 |
| 425 | Nephritis | 1.414931 |
| 426 | Respiratory Distress Syndrome, Adult | 1.414931 |
| 427 | Gastrointestinal Diseases | 1.414931 |
| 428 | Enterocolitis | 1.414931 |
| 429 | Drug abuse | 1.414931 |
| 430 | Bile duct carcinoma | 1.414931 |
| 431 | Urinary tract infection | 1.414931 |
| 432 | Abnormal mental state | 1.414931 |
| 433 | Spontaneous abortion | 1.414931 |
| 434 | Bitter | 1.414931 |
| 435 | Dysphonia | 1.414931 |
| 436 | Hyperlipidemia | 1.414931 |
| 437 | Cyst | 1.414931 |
| 438 | Postoperative Hemorrhage | 1.414931 |
| 439 | Rigor - Temperature-associated observation | 1.414931 |
| 440 | Cataract | 1.414931 |
| 441 | Growth retardation | 1.414931 |
| 442 | Dyspareunia | 1.414931 |
| 443 | Swallowing painful | 1.414931 |
| 444 | Flatulence | 1.414931 |
| 445 | Duane Retraction Syndrome | 1.414931 |
| 446 | Increase in blood pressure | 1.414931 |
| 447 | Pelvic abscess | 1.414931 |
| 448 | Avascular necrosis | 1.414931 |
| 449 | Deep Vein Thrombosis | 1.414931 |
| 450 | Endometrial Hyperplasia | 1.414931 |
| 451 | Bronchogenic Carcinoma | 1.414931 |
| 452 | Hypomagnesemia | 1.414931 |
| 453 | Telangiectasis | 1.414931 |
| 454 | Myocardial Ischemia | 1.414931 |
| 455 | Tic disorder | 1.414931 |
| 456 | Epilepsy | 1.414931 |
| 457 | Pneumonia | 1.334888 |
| 458 | Hypersensitivity | 1.333014 |
| 459 | Kidney Failure, Acute | 1.297148 |
| 460 | Cardiomyopathies | 1.297148 |
| 461 | Heart failure | 1.243081 |
| 462 | Cerebrovascular Disorders | 1.191788 |
| 463 | Heart valve disease | 1.191788 |
| 464 | Swollen Lymph Node | 1.191788 |
| 465 | Sleep Disorders | 1.191788 |
| 466 | Cytomegalovirus Infections | 1.191788 |
| 467 | Rheumatoid Arthritis | 1.191788 |
| 468 | Burn injury | 1.191788 |
| 469 | Vocal Cord Paralysis | 1.191788 |
| 470 | Splenomegaly | 1.191788 |
| 471 | Kidney Failure | 1.191788 |
| 472 | Ileus | 1.191788 |
| 473 | Myocarditis | 1.191788 |
| 474 | Aspiration pneumonitis | 1.191788 |
| 475 | Cystitis | 1.191788 |
| 476 | Myositis | 1.191788 |
| 477 | Encephalitis | 1.191788 |
| 478 | Hypercalcemia | 1.191788 |
| 479 | Tuberculosis | 1.191788 |
| 480 | Toxic Epidermal Necrolysis | 1.191788 |
| 481 | Acrodynia | 1.191788 |
| 482 | Bacterial Infections | 1.191788 |
| 483 | Hypokinesia | 1.191788 |
| 484 | Contusions | 1.191788 |
| 485 | Anger | 1.191788 |
| 486 | Liver Failure | 1.191788 |
| 487 | Bone Diseases | 1.191788 |
| 488 | Vaginal Hemorrhage | 1.191788 |
| 489 | Apnea | 1.191788 |
| 490 | Mental deterioration | 1.191788 |
| 491 | Hypokalemia | 1.191788 |
| 492 | Mycosis Fungoides | 1.191788 |
| 493 | Cholangitis | 1.191788 |
| 494 | Gastrointestinal obstruction | 1.191788 |
| 495 | Myalgia | 1.191788 |
| 496 | Henoch-Schoenlein Purpura | 1.191788 |
| 497 | Chronic Obstructive Airway Disease | 1.191788 |
| 498 | Blindness | 1.158998 |
| 499 | Thromboembolism | 1.142997 |
| 500 | Cerebrovascular accident | 1.096477 |
| 501 | Malignant Vaginal Neoplasm | 1.009466 |
| 502 | Peripheral edema | 1.009466 |
| 503 | Hyponatremia | 1.009466 |
| 504 | Osteoporosis | 1.009466 |
| 505 | Chronic Fatigue Syndrome | 1.009466 |
| 506 | Abdomen distended | 1.009466 |
| 507 | Liver damage | 1.009466 |
| 508 | Asthma | 1.009466 |
| 509 | Breast enlargement female | 1.009466 |
| 510 | Trismus | 1.009466 |
| 511 | Muscular Atrophy | 1.009466 |
| 512 | Hematoma | 1.009466 |
| 513 | Acidosis | 1.009466 |
| 514 | Sensory neuropathy | 1.009466 |
| 515 | Dehydration | 1.009466 |
| 516 | Nerve injury | 1.009466 |
| 517 | Burning sensation | 1.009466 |
| 518 | Lymphadenopathy | 1.009466 |
| 519 | Venous Thrombosis | 1.009466 |
| 520 | Fetal Alcohol Syndrome | 1.009466 |
| 521 | Wasting | 1.009466 |
| 522 | Cerebral Edema | 1.009466 |
| 523 | Blood Coagulation Disorders | 1.009466 |
| 524 | Calcinosis | 1.009466 |
| 525 | Depressed Level of Consciousness | 1.009466 |
| 526 | Colitis | 1.009466 |
| 527 | Alarm (not alarm reaction) | 1.009466 |
| 528 | Respiratory distress | 1.009466 |
| 529 | Headache | 1.009466 |
| 530 | Psoriasis | 1.009466 |
| 531 | Flushing | 1.009466 |
| 532 | Pain, Postoperative | 1.009466 |
| 533 | Airway Obstruction | 1.009466 |
| 534 | Mental blocking | 0.955399 |
| 535 | Coronary Artery Disease | 0.929423 |
| 536 | Alopecia | 0.929423 |
| 537 | Retinoblastoma | 0.855315 |
| 538 | Arthralgia | 0.855315 |
| 539 | Wound dehiscence | 0.855315 |
| 540 | Asthenia | 0.855315 |
| 541 | Endometrial adenocarcinoma | 0.855315 |
| 542 | Gigantism | 0.855315 |
| 543 | Teratoma | 0.855315 |
| 544 | Sedated state | 0.855315 |
| 545 | Aneurysm | 0.855315 |
| 546 | Hemolysis (disorder) | 0.855315 |
| 547 | Polymyalgia Rheumatica | 0.855315 |
| 548 | Bone Sarcoma | 0.855315 |
| 549 | Muscle Rigidity | 0.855315 |
| 550 | Mental disorders | 0.855315 |
| 551 | Proctitis | 0.855315 |
| 552 | Seminoma | 0.855315 |
| 553 | Falls | 0.855315 |
| 554 | Erythema | 0.786322 |
| 555 | Pulmonary aspiration | 0.764344 |
| 556 | Stomatitis | 0.721784 |
| 557 | Blood in stool | 0.721784 |
| 558 | Hairy Cell Leukemia | 0.721784 |
| 559 | Dysuria | 0.721784 |
| 560 | Malignant neoplasm of female breast | 0.721784 |
| 561 | Atrial Fibrillation | 0.721784 |
| 562 | Fibrillation | 0.721784 |
| 563 | Myeloproliferative disease | 0.721784 |
| 564 | Gastrointestinal Hemorrhage | 0.721784 |
| 565 | Mycoses | 0.721784 |
| 566 | Low pH | 0.721784 |
| 567 | Hyperglycemia | 0.721784 |
| 568 | Complex partial seizures | 0.721784 |
| 569 | Melena | 0.721784 |
| 570 | Hepatitis C | 0.721784 |
| 571 | Weakness | 0.686693 |
| 572 | Keratosis | 0.661159 |
| 573 | Kidney Diseases | 0.641741 |
| 574 | Neuroblastoma | 0.604001 |
| 575 | Primary Myelofibrosis | 0.604001 |
| 576 | Endometriosis | 0.604001 |
| 577 | Hypothyroidism | 0.604001 |
| 578 | Hypotension | 0.604001 |
| 579 | Pancreatic carcinoma | 0.604001 |
| 580 | Fatty Liver | 0.604001 |
| 581 | bite injury | 0.604001 |
| 582 | Renal tubular disorder | 0.604001 |
| 583 | Urethral Diseases | 0.604001 |
| 584 | Premenstrual syndrome | 0.604001 |
| 585 | Recurrent Carcinoma | 0.604001 |
| 586 | Peritonitis | 0.604001 |
| 587 | Pancreatitis | 0.604001 |
| 588 | Astrocytoma | 0.604001 |
| 589 | Anorexia | 0.582022 |
| 590 | Thrombosis | 0.567633 |
| 591 | Myelofibrosis | 0.549934 |
| 592 | Abdominal Pain | 0.532542 |
| 593 | Breath Holding | 0.49864 |
| 594 | Encephalopathies | 0.49864 |
| 595 | Acute leukemia | 0.49864 |
| 596 | Photopsia | 0.49864 |
| 597 | Back Pain | 0.49864 |
| 598 | Respiration Disorders | 0.49864 |
| 599 | Toxic nephropathy | 0.49864 |
| 600 | Weight Gain | 0.49864 |
| 601 | Lymphoma | 0.481147 |
| 602 | Hepatitis | 0.40333 |
| 603 | Ascites | 0.40333 |
| 604 | Neurotoxicity Syndromes | 0.40333 |
| 605 | Erectile dysfunction | 0.40333 |
| 606 | Penile Diseases | 0.40333 |
| 607 | Menopausal symptom | 0.40333 |
| 608 | Neuralgia | 0.40333 |
| 609 | Raised prostate specific antigen | 0.40333 |
| 610 | Proteinuria | 0.40333 |
| 611 | hearing impairment | 0.40333 |
| 612 | Graft-vs-Host Disease | 0.40333 |
| 613 | Benign neoplasm vagina | 0.40333 |
| 614 | Hematuria | 0.40333 |
| 615 | Multiple Organ Failure | 0.40333 |
| 616 | Cardiac Arrest | 0.380857 |
| 617 | Congenital Abnormality | 0.333126 |
| 618 | Urinary Incontinence | 0.316319 |
| 619 | Cachexia | 0.316319 |
| 620 | Anus Diseases | 0.316319 |
| 621 | Compression of spinal cord | 0.316319 |
| 622 | Heartburn | 0.316319 |
| 623 | Little's Disease | 0.241418 |
| 624 | Muscle hypotonia | 0.236276 |
| 625 | Purpura, Thrombotic Thrombocytopenic | 0.236276 |
| 626 | Bone pain | 0.236276 |
| 627 | Paralysed | 0.236276 |
| 628 | Confusion | 0.236276 |
| 629 | Icterus | 0.236276 |
| 630 | Gastrointestinal Neoplasms | 0.236276 |
| 631 | Arthritis | 0.236276 |
| 632 | Hyperactive behavior | 0.236276 |
| 633 | Dermatologic disorders | 0.236276 |
| 634 | Papilloma | 0.236276 |
| 635 | Lymphopenia | 0.236276 |
| 636 | Hemoptysis | 0.236276 |
| 637 | Shock | 0.198536 |
| 638 | Deglutition Disorders | 0.186266 |
| 639 | Hydronephrosis | 0.162168 |
| 640 | Patent ductus arteriosus | 0.162168 |
| 641 | Hot flushes | 0.162168 |
| 642 | Intestinal Obstruction | 0.162168 |
| 643 | Sexual Dysfunction | 0.162168 |
| 644 | MYELODYSPLASTIC SYNDROME | 0.162168 |
| 645 | Endocrine System Diseases | 0.162168 |
| 646 | Sting Injury | 0.162168 |
| 647 | Cicatrix | 0.093175 |
| 648 | Liver Cirrhosis | 0.093175 |
| 649 | Drowsiness | 0.093175 |
| 650 | Pathologic Neovascularization | 0.093175 |
| 651 | Myeloid Leukemia | 0.028637 |
| 652 | Cardiovascular Diseases | 0.028637 |
| 653 | Palmar-plantar erythrodysesthesia syndrome | 0.028637 |
| 654 | Uterine Diseases | 0.028637 |
| 655 | Ovarian Carcinoma | 0.028637 |
| 656 | Respiratory Failure | 0.028637 |
| 657 | Delirium | 0.028637 |
| 658 | Spinal Cord Diseases | -0.00213 |
| 659 | Physiologic tolerance | -0.01717 |
| 660 | Sleep disturbances | -0.03199 |
| 661 | Heart Diseases | -0.03199 |
| 662 | Nausea | -0.04174 |
| 663 | Malignant neoplasm of brain | -0.06098 |
| 664 | Acquired Immunodeficiency Syndrome | -0.08915 |
| 665 | Gallbladder Carcinoma | -0.08915 |
| 666 | Dermatitis, Phototoxic | -0.08915 |
| 667 | Xerostomia | -0.08915 |
| 668 | Carpal Tunnel Syndrome | -0.08915 |
| 669 | Suicide | -0.08915 |
| 670 | Sepsis | -0.08915 |
| 671 | Abscess | -0.08915 |
| 672 | Herpes Simplex Infections | -0.14321 |
| 673 | Malaise | -0.16919 |
| 674 | Leukopenia | -0.16919 |
| 675 | Sleeplessness | -0.19451 |
| 676 | Excitation | -0.19451 |
| 677 | Kidney Failure, Chronic | -0.2192 |
| 678 | Premature Birth | -0.26683 |
| 679 | Lymphoma, Non-Hodgkin | -0.28982 |
| 680 | Esophagitis | -0.28982 |
| 681 | Constipation | -0.28982 |
| 682 | Vomiting | -0.30485 |
| 683 | Cessation of life | -0.31098 |
| 684 | Diabetes Mellitus | -0.31229 |
| 685 | Pleural Mesothelioma | -0.33427 |
| 686 | Peripheral Nervous System Diseases | -0.37683 |
| 687 | Skin carcinoma | -0.37683 |
| 688 | Hepatobiliary Disorder | -0.41765 |
| 689 | Lymphedema | -0.41765 |
| 690 | Thrombocytopenia | -0.43745 |
| 691 | Thyroid carcinoma | -0.45687 |
| 692 | Hypertensive disease | -0.49461 |
| 693 | Malignant neoplasm of mouth | -0.53098 |
| 694 | Liver diseases | -0.56607 |
| 695 | Surgical intervention (finding) | -0.56607 |
| 696 | Skin Basal Cell Carcinoma | -0.56607 |
| 697 | Seizures | -0.59997 |
| 698 | Malignant neoplasm of testis | -0.63276 |
| 699 | Lymphoid leukemia | -0.63276 |
| 700 | Multiple Myeloma | -0.63276 |
| 701 | Dermatitis | -0.63276 |
| 702 | Atrial Premature Complexes | -0.66451 |
| 703 | leukemia | -0.71775 |
| 704 | Ovarian Diseases | -0.72514 |
| 705 | Fatigue | -0.72933 |
| 706 | Obesity | -0.75412 |
| 707 | Malignant neoplasm of larynx | -0.75412 |
| 708 | Exhaustion | -0.78229 |
| 709 | Hemorrhage | -0.78229 |
| 710 | Fear (Mental Process) | -0.86234 |
| 711 | Thyroid Diseases | -0.86234 |
| 712 | Cold Sensation | -0.88765 |
| 713 | Liver neoplasms | -0.88765 |
| 714 | Extravasation | -0.91235 |
| 715 | Recurrent tumor | -0.92447 |
| 716 | Anemia | -0.93644 |
| 717 | Renal carcinoma | -0.98296 |
| 718 | Pregnancy | -0.98296 |
| 719 | Malignant neoplasm of thyroid | -0.98296 |
| 720 | Neuropathy | -1.09059 |
| 721 | Edema | -1.1108 |
| 722 | Peeling of skin | -1.1108 |
| 723 | Hypoxia | -1.1306 |
| 724 | Carcinoma in situ of larynx | -1.15002 |
| 725 | Benign neoplasm of larynx | -1.15002 |
| 726 | Inflammatory disease of mucous membrane | -1.16907 |
| 727 | Neutropenia | -1.18776 |
| 728 | Dental caries | -1.19698 |
| 729 | Pain | -1.22157 |
| 730 | Virus Diseases | -1.24183 |
| 731 | Weight decreased | -1.25922 |
| 732 | Autoimmune Diseases | -1.27631 |
| 733 | Sarcoma | -1.28475 |
| 734 | Mental Depression | -1.30965 |
| 735 | Rectal Diseases | -1.43288 |
| 736 | Fever | -1.46427 |
| 737 | Urologic Diseases | -1.46614 |
| 738 | Malignant neoplasm of endometrium | -1.51626 |
| 739 | Malignant neoplasm of gastrointestinal tract | -1.55548 |
| 740 | melanoma | -1.56822 |
| 741 | Malignant neoplasm of skin | -1.67611 |
| 742 | Urinary Retention | -1.86221 |
| 743 | Malignant neoplasm of liver | -1.86221 |
| 744 | Infection | -1.92173 |
| 745 | Malignant neoplasm of pancreas | -2.11143 |
| 746 | Anxiety | -2.13325 |
| 747 | Stomach Carcinoma | -2.21936 |
| 748 | Cervix carcinoma | -2.25502 |
| 749 | Aggressive behavior | -2.27395 |
| 750 | Esophageal carcinoma | -2.30472 |
| 751 | Liver carcinoma | -2.36926 |
| 752 | Carcinoma of bladder | -2.47177 |
| 753 | Esophageal Diseases | -2.73611 |
| 754 | Rectal Carcinoma | -2.97643 |
| 755 | Prostatic Diseases | -3.54265 |
